# Inborn Errors of Immunity on the Island of Ireland — a Cross-Jurisdictional UKPID/ESID Registry Report

**DOI:** 10.1007/s10875-022-01274-w

**Published:** 2022-05-23

**Authors:** Paul Ryan, Vyanka Redenbaugh, Jayne McGucken, Gerhard Kindle, Lisa A. Devlin, Tanya Coulter, Matthew S. Buckland, Mikko R. J. Seppänen, Niall P. Conlon, Conleth Feighery, J. David M. Edgar

**Affiliations:** 1 St. James’s Hospital and Trinity College Dublin, Dublin, Republic of Ireland; 2 Belfast Health and Social Care Trust, Belfast, Northern Ireland; 3 Institute for Immunodeficiency, Center for Chronic Immunodeficiency, Medical Centre, Faculty of Medicine, University of Freiburg, Freiburg, Germany; 4 Molecular and Cellular Immunology Section, Immunity and Inflammation Department, UCL Great Ormond Street Institute of Child Health, London, England; 5 Rare Disease and Pediatric Research Centers, University of Helsinki and Helsinki University Hospital, Helsinki, Finland

**Keywords:** Immunodeficiency, IEI (inborn errors of immunity), prevalence, UKPID, ESID, registry

## Abstract

The epidemiology of inborn errors of immunity (IEI) in the Republic of Ireland was first published in 2005 but has not been updated since. IEI prevalence data from Northern Ireland was last published in 2018. Using data from the United Kingdom Primary Immune Deficiency (UKPID) and European Society for Immunodeficiencies (ESID) registries, we reviewed all registered cases of IEI affecting adult patients ≥ 18 years of age from the two largest immunology specialist centres in Northern Ireland and the Republic of Ireland, respectively and calculated the combined minimum adult prevalence of IEI on the island of Ireland for the first time. We also recorded data pertaining to presenting symptoms of IEI, diagnostic delay, immunoglobulin data, and genetic testing, as well as briefly reporting data pertaining to secondary immunodeficiency in both countries. As of 1 May 2020, we identified a minimum adult IEI prevalence in Ireland of 8.85/100,000 population.

## Introduction

Inborn errors of immunity (IEI), also referred to as primary immunodeficiencies (PID), are a heterogeneous group of disorders caused mainly by germline mutations resulting in loss of expression, loss-of-function, or gain-of-function of an encoded protein [1]. IEI most commonly presents as an increased susceptibility to infection, but there is growing recognition that IEI frequently presents with immune dysregulation, syndromic features, and/or malignancy, in tandem or in isolation. In 2020, the International Union of Immunological Societies (IUIS) published an updated list of 430 distinct single-gene IEIs underlying phenotypes as diverse as infection susceptibility, allergy, autoimmunity, autoinflammation, and malignancy [2]. Since that publication, 26 additional monogenic gene defects have been identified, adding to the ever-growing array of novel inborn errors of immunity [3].

While individually rare, IEI might collectively be more common than assumed [4]. Reported prevalence has ranged from 1:16,000 to 1:50,000 [5], but, with the ongoing discovery of novel inborn errors of immunity and improved definition of clinical phenotypes, collective prevalence might be closer to 1:5,000 [6]. Nevertheless, the small number of patients cared for by individual clinicians and centres poses a challenge to early diagnosis, care, and research. The last report on IEI in a solely Irish population was in 2005 and reported a prevalence of 2.9/100,000 population in the Republic of Ireland [7].

Collaborative national and international registries like United Kingdom Primary Immune Deficiency (UKPID) and the European Society for Immunodeficiencies (ESID) registry have tried to circumnavigate the individual rarity of these conditions by collecting and reporting data from all over Europe, acting as a pooled source of epidemiologic, clinical, and genetic information.

The Regional Immunology Service in Northern Ireland, based in The Royal Hospitals, Belfast Health and Social Care Trust, contributes to both UKPID and ESID for both paediatric and adult populations. It is the only immunology specialist centre in Northern Ireland. While there are several immunology specialist centres in the Republic of Ireland, St. James’s Hospital (SJH) in Dublin is the only immunology service in Ireland that currently contributes to the ESID registry. Both centres lie on the island of Ireland, have close collegiate ties, and are of a similar size.

This report is the first collaborative report on IEI affecting an adult population ≥ 18 years of age from the two largest immunology centres on the island of Ireland.

## Methods

A retrospective analysis of the ESID and UKPID registries pertaining to all adult patients ≥ 18 years of age with IEI attending both centres in Belfast and Dublin was carried out, using 1 May 2020 as the time point for prevalence calculations. Multicentre Research Ethics (MREC) approval was obtained in 2004 for the ESID online database (MREC number: 04/MRE07/68), with approvals amended to reflect the establishment of a UK-based database. Local ethics approval to contribute to the ESID database was obtained in SJH, Dublin, in 2019 (REC: 2019–10 List 39(2)). All patients included in the ESID registry provided their consent in writing in compliance with General Data Protection Regulation (GDPR).

Prevalence data were calculated using national population data and/or estimated catchment population data. As the Regional Immunology Service serves the entire population of Northern Ireland, minimum prevalence data for Northern Ireland were calculated using the latest available population estimate of 1,893,667 people from the Northern Ireland Statistics and Research Agency (NISRA) in June 2019 [8]. SJH, Dublin, provides services for adult patients with immunodeficiency disorders from south Dublin and southern districts of the Republic of Ireland. While there is no formalised catchment area, the regional population from which referrals are received is 2,241,639 based on the latest available community healthcare organisation (CHO) data [9]. We have therefore reported three different sets of prevalence data: minimum prevalence data for Northern Ireland, minimum prevalence data for the Republic of Ireland using the estimated catchment population of SJH, Dublin, and combined all-Ireland minimum prevalence data for the two centres. In all instances, prevalence data calculations were rounded up or down to the nearest two decimal places.

To ensure that only adult patients ≥ 18 years of age were included, a year of birth of 2001 was used as the cut-off for inclusion. Any patient with a year of birth of 2002 or above was excluded from the report.

Diagnostic delay was defined as the time in years from the first presenting symptom(s) to the time of the first clinical diagnosis of IEI, as documented on the UKPID and/or ESID registries. When the year of onset of the first symptom(s) was documented to be the same as the year of the first clinical diagnosis of IEI, diagnostic delay was said to be 0 years.

The patients with secondary immunodeficiencies, while included in the UKPID registry but not in the ESID registry, were included in this report to demonstrate their contribution to national prevalence and immunoglobulin data, as well as their contribution to the workload of the clinical immunology teams at both sites. Local electronic patient record (EPR) was used to collect data on secondary immunodeficiency at SJH, while the UKPID registry was used to collect similar data from Northern Ireland.

## Results

As of 1 May 2020, 395 adult patients with IEI had attended specialist immunology services at both centres and were registered on the ESID and UKPID registries, 366 (92.66%) of whom were under active follow-up. This equates to a minimum IEI prevalence of 7.09/100,000 population in the Republic of Ireland, 10.93/100,000 population in Northern Ireland, and a combined Ireland minimum prevalence of 8.85/100,000 population. Prevalence data for the 2019 updated International Union of Immunological Societies (IUIS) classification categories [1] are shown in Table 1.

**Table 1:** Prevalence data for the updated International of Union Immunological Societies (IUIS) classification categories as of 1 May 2020

IUIS Classification 2019	*n* (all patients registered)	*n* (alive and under follow-up)	Minimum Republic of Ireland prevalence/100,000 population	Minimum Northern Ireland prevalence/100,000 population	Minimum Combined prevalence/100,000 population
Immunodeficiencies affecting cellular or humoral immunity	3	3	0.04	0.11	0.07
Combined immunodeficiencies with associated or syndromic features	18	17	0.54	0.26	0.41
Predominantly antibody deficiencies	258	243	4.42	7.60	5.88
Diseases of immune dysregulation	13	12	0.27	0.32	0.29
Congenital defects of phagocytic number or function	7	7	0.13	0.21	0.17
Defects in intrinsic and innate immunity	4	4	0.04	0.16	0.10
Autoinflammatory disorders	14	12	0.31	0.26	0.29
Complement deficiencies	56	48	1.20	1.11	1.16
Bone marrow failure	0	0	0.00	0.00	0.00
Phenocopies of inborn errors of metabolism	13	11	0.09	0.48	0.27
Unclassified or unknown	9	9	0.04	0.42	0.22
Total	395	366	7.09	10.93	8.85

Fifteen patients with IEI (3.79%) had died since being registered on the ESID registry, and 14 (3.54%) had been lost to follow-up.

Antibody deficiencies made up the largest group of patients, accounting for 258 patients registered (65.31%). The most commonly reported IEI was common variable immunodeficiency (CVID), accounting for 131 patients (33.16%). The second most common IEI recorded was hereditary angioedema (HAE) (*n* = 48, 12.15%). Unclassified antibody deficiency (*n* = 34, 8.61%), selective IgA deficiency (*n* = 33, 8.35%), agammaglobulinaemia (*n* = 28, 7.09%), and deficiency of specific IgG (*n* = 14, 3.54%) were the next most common diagnoses. Using the estimated catchment populations of both centres, the minimum combined prevalence of CVID was 2.93/100,000, HAE 1.04/100,000, unclassified antibody deficiency 0.80/100,000, selective IgA deficiency 0.75/100,000, and agammaglobulinaemia 0.68/100,000.

There were 206 (52.15%) females and 189 (47.85%) males with IEI.

Including index cases, 64 (16.20%) cases of IEI were identified as familial cases; the majority of familial cases were agammaglobulinaemia (31.25%, *n* = 20) and HAE (21.88%, *n* = 14).

There were no documented cases of consanguinity.

One hundred and thirty-eight (34.94%) patients with IEI had undergone genetic testing, in whom 58 disease-causing variants or likely disease-causing variants were identified (14.68% of all the patients, or 42.03% of those genetically tested). Pertaining to CVID, 45 of 131 patients (34.35%) had undergone genetic testing. No disease-causing variants were found in 36 patients (80%); 9 patients (20%) were found to have disease-causing variants or likely disease-causing variants. While these 9 patients are still recorded on the UKPID and ESID databases as having CVID (and have therefore been recorded in this report as such), their respective diagnoses of CVID may be amended or changed in future reports.

Of patients with agammaglobulinaemia, 22 of the 28 registered patients had undergone genetic testing (78.57%); all 22 had defects in *BTK*.

Six patients with chronic granulomatous disease (CGD) were registered, 83.33% (*n* = 5) of whom had a proven genetic defect, with disease-causing variants in *CYBB* gene encoding the GP91-phox protein accounting for 3 cases; disease-causing variants in *CYBA* gene encoding P22-phox protein (*n* = 1) and in *NCF1* gene encoding P47-phox (*n* = 1) accounted for the remaining cases.

Of the 395 patients registered with IEI, 336 (85.06%) had data recorded in relation to presenting symptom(s). The main presenting symptom was infection-related (61.52%%, *n* = 243), in isolation or in combination with other signs and symptoms, followed by immune dysregulation (11.89%, *n* = 47). While infection and immune dysregulation sometimes occurred in combination at presentation (4.30%, *n* = 17), there were 28 patients (7.09%) with IEI who presented with immune dysregulation alone in the absence of a history of infection. Eight patients presented with syndromic features (2.03%); 1 patient presented with malignancy (0.25%); and 13 patients had no symptoms of IEI at the time of initial evaluation (3.87%). Presenting symptom was not recorded for 59 patients (14.94%). Incidental findings on laboratory testing led to a diagnosis of selective IgA deficiency in 2 cases (0.51%), and a family study led to the identification of 1 case of complement component 7 deficiency (0.25%).

Angioedema was the presenting feature in 42 patients (10.63%), all of whom had a diagnosis of HAE or acquired angioedema.

A total of 82 patients with IEI were recorded to have bronchiectasis (20.76%), a major cause of morbidity amongst patients with IEI. Four patients with IEI (1.01%) underwent splenectomy, 2 of whom had a diagnosis of CVID, 1 of whom had early onset multi-organ autoimmune disease, and 1 of whom had a diagnosis of proline/serine/threonine phosphatase-interacting protein 1 (PSTPIP1) deficiency. No patients were recorded to have undergone gene therapy.

Diagnostic delay — defined as the time delay in years between documentation of onset of first symptom(s) and documentation of the first clinical diagnosis of immunodeficiency — was calculated for each condition when data was available. A total of 253 patients had data recorded to permit this calculation. Median diagnostic delay for all the patients with IEI was 3 years (IQR = 1–8.5 years). Median diagnostic delay for predominantly antibody deficiencies was 3 years (IQR = 1–8 years), and for CVID was 4 years (IQR = 1–7 years). Median diagnostic delay was much longer for diseases of immune dysregulation (4.5 years), diseases of intrinsic and innate immunity (5 years), and autoinflammatory disorders (25 years).

Notable lengthy diagnostic delays for individual cases included: 3 cases of familial cold autoinflammatory syndrome (34, 40 and 65 years respectively); 1 case of chronic mucocutaneous candidiasis (43 years); 1 case of PSTPIP1 (proline/serine/threonine phosphatase-interacting protein 1) deficiency (34 years); 1 case of Mendelian susceptibility to mycobacterial diseases (MSMD) (33 years); 1 case of tumour necrosis factor receptor–associated periodic syndrome (TRAPS) (33 years); 1 case of activated phosphoinisitide 3-kinase delta syndrome (APDS) (25 years); 2 cases of mevalonate kinase deficiency (17 and 12 years, respectively); and 1 case of chronic granulomatous disease (CGD) (15 years).

A total of 221 patients with IEI were recorded to have received immunoglobulin replacement therapy (47.84% of the 462 patients with IEI registered at both centres). A further 60 patients with secondary immunodeficiency received immunoglobulin replacement therapy at both centres. At SJH, Dublin, there was a large transition to home therapy during the COVID-19 pandemic, with 69.23% of patients receiving immunoglobulin therapy at home (72 of the 104 patients receiving immunoglobulin at SJH). In Northern Ireland, a total of 177 patients received immunoglobulin replacement therapy: 62 (35.03%) were on home therapy; 25 (14.12%) attended outreach centres in their local area; and 90 (50.85%) received immunoglobulin therapy in the hospital setting.

A total of 11 patients received haematopoietic stem cell transplantation (HSCT). Two patients received matched-related HSCT; 5 patients received matched-unrelated peripheral blood stem cell transplantation; the sources of 4 HSCTs were not recorded. All the 11 patients were alive and under follow-up.

A total of 67 patients with secondary immunodeficiency attended both sites, of whom 61 were under active follow-up (25 in SJH and 36 in Northern Ireland). Minimum prevalence of secondary immunodeficiency was 1.12/100,000 in SJH and 1.90/100,000 in Northern Ireland, with a combined minimum prevalence of 1.48/100,000, making it second only to CVID in minimum prevalence. Six patients with secondary immunodeficiency (8.96%) had died since being registered on UKPID. Forty-two females and 25 males were treated at both centres for secondary immunodeficiency. Median diagnostic delay was 1 year (IQR 1–3 years). A total of 60 patients with secondary immunodeficiency received immunoglobulin replacement therapy at both centres (representing 21.35% of the 281 patients receiving treatment).

The vast majority of patients with secondary immunodeficiency presented with infection-related symptoms (95.52%). A small minority presented with immune dysregulation (2.99%) or other symptoms (1.49%).

Data regarding the underlying cause of secondary immunodeficiency was not readily available for patients in Northern Ireland on the UKPID registry. In the SJH cohort, 15 patients had steroid-induced hypogammaglobulinaemia; other patients had secondary immunodeficiency as a consequence of chemotherapy (*n* = 5); rituximab (*n* = 1); and clozapine (*n* = 1); the remaining underlying aetiologies were not readily available.

## Discussion

Registries have played a critical part in collating and reporting our collective experience and understanding of IEI. Many European countries contribute to their own national registries. The French National Reference Centre of Primary Immunodeficiencies (CEREDIH) was established in 2005. The UKPID registry was founded in 2008, and the German PID-NET registry was launched in 2009.

SJH, Dublin, is the largest adult hospital in Republic of Ireland, with a capacity of 1010 beds and an estimated catchment population for immunology referrals of 2,241,639 people. The Regional Immunology Service in Belfast serves a catchment population of 1,893,667 people and operates from within The Royal Hospitals, Belfast Health and Social Care Trust, which has a combined capacity of 1317 adult beds. Both centres offer specialist immunology services in both primary and secondary immunodeficiencies, allergy, and vasculitis, and have close collegiate ties. Both centres contribute to the ESID registry, and the Regional Immunology Service in Belfast also contributes to the UKPID registry.

In 2005, Abuzakouk and Feighery [7] published the first report on IEI in the Republic of Ireland. Being based in SJH, Dublin, the authors drew on their own local IEI database to draft the report and also collected data using a questionnaire that was submitted to hospitals nationally. Their data included both adult and paediatric patients. They identified a total of 115 patients with IEI. Antibody deficiencies (*n* = 52) made up the majority, comprising 28 cases of common variable immunodeficiency (CVID) and 25 cases of X-linked agammaglobulinaemia. Complement deficiency was the second most frequently established diagnosis with 32 cases. Using these data, the authors calculated a minimum Republic of Ireland national prevalence of 2.9/100,000 population. The authors acknowledged that their data likely underestimated the true national prevalence of IEI at the time due to under recognition of IEI in centres without immunology specialist services and the fact that many patients may not have been captured using a questionnaire method of data collection.

The first report on IEI from the Regional Immunology Service, Belfast, was published by Edgar et al. in 2014 [10] as part of a report of the first 4 years of activity of the UKPID registry. Eighty-two patients, both adult and paediatric, were registered at the Royal Hospitals at the time. Using a reported population of Northern Ireland of 1,840,500 people at the time [11], this equated to a minimum IEI prevalence in Northern Ireland of 4.46/100,000 population.

As such, the reported prevalence of IEI in both Northern Ireland and Republic of Ireland has increased significantly since the publication of these first reports. In the Republic of Ireland, the minimum prevalence of IEI has risen from 2.9/100,000 in 2005 to 7.09/100,000 in 2020. In Northern Ireland, IEI prevalence has increased from 4.46/100,000 in 2014 to 1093/100,000 in 2020. This rise in prevalence is probably due to a combination of better diagnostics, more robust referral pathways, and a wider recognition of the importance of recording and contributing data to international IEI registries.

### IEI Prevalence

As of 1 May 2020, the minimum adult prevalence of IEI was 8.85/100,000 in Ireland. To put this in context, the Irish prevalence of motor neuron disease is 3.3–4.82/100,000 [12, 13]; haemophilia B (in patients ≥ 17 years of age) is 7.92/100,000 population [14]; and cystic fibrosis (in patients ≥ 18 years of age) is 14.99/100,000 [15].

Most countries publish data in combined paediatric and adult datasets. For example, the latest minimum IEI prevalence in the UK of 5.90/100,000 includes both adult and paediatric patients [16]. Other countries have published combined adults and paediatric datasets while also providing the number of adult patients ≥ 18 years of age with IEI in their reports, allowing us to extrapolate an adult minimum prevalence based on that country’s population. For example, Germany has not only reported a combined adult and paediatric minimum IEI prevalence of 2.72/100,000, but also published the number of living adult patients with IEI ≥ 18 years of age (*n* = 1229) and the total population of Germany at the time of the report (*n* = 82,576,900), implying a minimum adult IEI prevalence in Germany of 1.49/100,000 [17]. Similarly, the Swiss National Registry for Primary Immunodeficiencies reported a living adult IEI patient number of 229 patients ≥ 18 years of age, allowing us to infer a minimum adult IEI prevalence of 2.85/100,000 (using the 2014 Swiss population of 8.04 million in their report) [18]. France estimates a total minimum IEI prevalence of 11/100,000 and an adult minimum IEI prevalence of 6.7/100,000 [19].

The Irish adult minimum IEI prevalence is, therefore, higher than that of the UK, France, Germany, and Switzerland when compared to both adult-only or combined adult, and paediatric prevalence data.

### Presenting Symptom(s)

Traditional teaching has emphasised the concept of recurrent infection as the hallmark of IEI. It is now recognised that autoimmune and autoinflammatory manifestations (referred to collectively as immune dysregulation) frequently herald IEI. In a retrospective review of the French IEI registry, 26.2% of patients had demonstrated manifestations of immune dysregulation at some point in their lifetime [20]. A recent report on the presenting manifestations in 16,486 patients with IEI found that 68% of patients presented with infections only; 9% presented with immune dysregulation only; and 9% with a combination of both [21]. In our report, only 220 of 395 patients with IEI (55.70%) presented exclusively with infection-related symptoms; 243 (61.52%) presented with infection and/or other symptoms; 17 patients (4.30%) presented with infection and immune dysregulation simultaneously; and 28 patients (7.09%) presented with manifestations of immune dysregulation alone in the absence of a history of infection. These data imply that an infection-focused approach to diagnosing IEI in Ireland would miss up to 38.48% of cases of IEI.

### Diagnostic Delay

Diagnostic delay can affect outcome negatively in IEI by delaying the implementation of essential treatment [22–26]. Median diagnostic delay for all the patients with IEI was 3 years (IQR = 1–8.5 years); in CVID 4 years (IQR = 1–7 years); and in agammaglobulinaemia 1 year (IQR = 0–3 years).

It is worth pointing out that lengthy diagnostic delays occasionally occurred with good explanation. For example, a diagnostic delay of 33 years in a case of Mendelian susceptibility to mycobacterial diseases (MSMD) was due simply to the fact that this condition was first described when the patient was in their thirties. A diagnosis of CVID was changed to activated PI3K-delta syndrome (APDS) after its first description in 2015 and when genetic testing became available.

Diagnostic delay is often defined differently, making comparison between reports difficult. For example, the French IEI registry (CEREDIH) defines diagnostic delay as the time between birth and the first clinical diagnosis of IEI [27], giving a median diagnostic delay for CVID of 6 years and median diagnostic delay for agammaglobulinaemia of 1 year. On the other hand, the German National Registry of Primary Immunodeficiencies defined diagnostic delay as the time elapsed between the first presenting symptom(s) and the date of either genetic or clinical diagnosis [17]. The Swiss National Registry for Primary Immunodeficiencies used a similar definition [18]. This is the definition that we too have adopted for this report, because the ESID registry records data regarding the date of first symptoms of IEI and the date of clinical diagnosis. Comparing data using this shared definition of diagnostic delay, the median diagnostic delay for CVID in Ireland was 4 years, in Germany 3 years, and in Switzerland 5.95 years. Data pertaining to diagnostic delay for other individual disorders was not readily available.

### Strengths and Limitations

The main strength of our current dataset is that it includes registered data from the ESID registry for the first time to calculate minimum IEI prevalence data for the Republic of Ireland, rather than relying on local databases and national surveys.

The main limitation of these data is that they still likely significantly underestimate the true prevalence of IEI in Ireland, as no immunology specialist centres in the Republic of Ireland other than SJH in Dublin currently contribute data to the ESID registry. Indeed, we know from informal estimates of the numbers of patients attending other hospitals in the Republic of Ireland that there are an additional 558 patients with IEI that have yet to be registered on the ESID registry. These informal data include: 139 patients with CVID; 109 patients with Specific IgG deficiency (SPAD); 60 patients with unclassified antibody deficiency; 42 patients with HAE; and 6 patients with agammaglobulinaemia [Keogan M, Tormey V, Leahy T, O’ Leary, P. Personal communication]. Using these data in combination with registered ESID data, there are at least 732 patients with IEI in the Republic of Ireland, suggesting a prevalence of 14.71/100,000 population in the Republic of Ireland, and a combined IEI prevalence on the island of Ireland of 13.87/100,000 population.

## Conclusions

This is the first report on IEI on the island of Ireland to include immunology specialist centres in both Northern Ireland and the Republic of Ireland. The minimum prevalence of PID in adults ≥ 18 years is 1093/100,000 in Northern Ireland and 7.09/100,000 in the Republic of Ireland, with a combined minimum IEI prevalence on the island of Ireland of 8.85/100,000, a figure that is higher than that of many other European countries. This figure still significantly underestimates the true prevalence of IEI in Ireland, as the current report only includes registered data from two immunology specialist centres on the island.

It is our hope that more immunology specialist services in the Republic of Ireland will start to contribute to the ESID registry so that future reports will more accurately represent the true epidemiology of IEI in Ireland. This report will be used to inform future public health policy and advocate for the expansion of immunology specialist services in Ireland.

## Data Availability

All the data presented in this manuscript are made available in the provided Table.
