# Correction to: Inborn Errors of Immunity on the Island of Ireland — a Cross‑Jurisdictional UKPID/ESID Registry Report

**DOI:** 10.1007/s10875-022-01306-5

**Published:** 2022-06-22

**Authors:** Paul Ryan, Vyanka Redenbaugh, Jayne McGucken, Gerhard Kindle, Lisa A. Devlin, Tanya Coulter, Matthew S. Buckland, Mikko R. J. Seppänen, Niall P. Conlon, Conleth Feighery, J. David M. Edgar

**Affiliations:** 1 St. James’s Hospital and Trinity College Dublin, Dublin, Republic of Ireland; 2 Belfast Health and Social Care Trust, Belfast, Northern Ireland; 3 Institute for Immunodeficiency, Center for Chronic Immunodeficiency, Medical Centre, Faculty of Medicine, University of Freiburg, Freiburg, Germany; 4 Molecular and Cellular Immunology Section, Immunity and Inflammation Department, UCL Great Ormond Street Institute of Child Health, London, England; 5 Rare Disease and Pediatric Research Centers, University of Helsinki and Helsinki University Hospital, Helsinki, Finland

**Correction to: Journal of Clinical Immunology**

**https://doi.org/10.1007/s10875-022-01274-w**

Due to typesetting mistake, the prevalence of 10.93/100,000 in Northern Ireland was mistakenly presented as "1093/100,000".

The original version has been corrected.

